# Refracture or death after osteoporotic fracture: five-year competing-risk and multistate trajectories

**DOI:** 10.3389/fendo.2026.1905993

**Published:** 2026-09-16

**Authors:** Yanjun Zhang, Bo Li, Zhechen Li, Zetong Zheng, Yinglun Zhao, Xinwei Zhang, Qian Lu, Zengtong Zhao, Peiyu Wang, Libin Cui, Yan Zhang, Menglong Li, Liang Liu

**Affiliations:** 1 Department of Bone Center, Beijing Luhe Hospital affiliated to Capital Medical University, Beijing, China; 2 Clinical Medical College, Capital Medical University, Beijing, China; 3 Queen Mary School, Nanchang University, Nanchang, Jiangxi, China; 4 Evidence-based Medicine Center, Beijing Luhe Hospital, Capital Medical University, Beijing, China

**Keywords:** competing risk, mortality, multistate model, osteoporotic fracture, refracture

## Abstract

**Background:**

Secondary fracture prevention is a priority after osteoporotic fracture, but patients remain at substantial risk of both refracture and death. Because death precludes a subsequently observed refracture, treating it as non-informative censoring can overestimate absolute refracture risk. We therefore used competing-risk and multistate methods to characterize the 5-year trajectories of first recorded hospitalized refracture and death after osteoporotic fracture.

**Methods:**

We conducted a retrospective secondary analysis of a de-identified public longitudinal dataset comprising 4,782 patients aged ≥50 years who were hospitalized for osteoporotic fracture between 2017 and 2024. Follow-up began at discharge from the index hospitalization, and the primary analysis included patients with a survival time of ≥30 days after discharge. First recorded hospitalized refracture was the target event, and death before refracture was treated as a competing event. Cumulative incidence functions, cause-specific Cox regression, Fine–Gray regression, and Aalen–Johansen estimation were used to quantify event occurrence, assess covariate associations, and estimate four-state occupation probabilities.

**Results:**

Thirty-one patients died within 0–26 days after discharge and were excluded, leaving 4,751 patients in the primary cohort. Among these patients, 258 experienced first recorded hospitalized refracture as the first event, 487 died before recorded refracture, and 4,006 were censored before either event. At 5 years, the cumulative incidence was 8.63% (95% CI, 7.51–9.75%) for recorded hospitalized refracture and 15.57% (95% CI, 14.12–17.03%) for competing death. The corresponding Aalen–Johansen state probabilities were 75.80% for being alive without recorded refracture, 6.76% for being alive after recorded refracture, 15.57% for death without recorded refracture, and 1.88% for death after recorded refracture. Forty-seven patients had an observed refracture-to-death transition within 5 years, and the small event count limited the precision of estimates for this state.

**Conclusion:**

Among patients with a survival time of ≥30 days after discharge following osteoporotic fracture, the 5-year risk of death before recorded refracture exceeded that of first recorded hospitalized refracture. Competing-risk and multistate methods provided complementary estimates of absolute event risks and subsequent clinical trajectories. These findings are conditional on early post-discharge survival and hospitalization-based outcome capture, which may underestimate the overall refracture burden, particularly for vertebral fractures managed outside hospital settings.

## Introduction

Osteoporotic fracture is a sentinel clinical event that identifies individuals with underlying skeletal fragility and increased risks of subsequent fracture, disability, healthcare utilization, and death (1–5). Although secondary fracture prevention is central to osteoporosis care (1, 2), post-fracture prognosis is determined by more than refracture alone. In older and comorbid populations, death is common and precludes any subsequent refracture. Treating such deaths as non-informative censoring may therefore overestimate the absolute occurrence of refracture, particularly over extended follow-up.

Kaplan–Meier estimates that censor competing deaths do not represent the observed cumulative probability of refracture when mortality is substantial. Cox models that censor competing deaths can validly estimate cause-specific associations but do not directly estimate absolute refracture probability. Cause-specific Cox models quantify covariate associations with the instantaneous hazard of refracture among patients who remain alive and refracture-free, whereas Fine–Gray models quantify associations with the subdistribution hazard underlying the cumulative incidence function (6–8). These estimands are complementary but answer different clinical questions. Multistate models extend this framework by quantifying transitions among clinically meaningful states, including being alive without refracture, alive after refracture, dead without refracture, and dead after refracture. Recent studies of older fracture populations have demonstrated the value of integrating competing-risk and multistate approaches when refracture and mortality are evaluated simultaneously (9, 10).

A recently published *Scientific Data* dataset contains patient-level demographic, fracture, treatment, comorbidity, mortality, refracture, and prescription information (11, 12). We therefore aimed to estimate the 5-year cumulative incidences of first recorded hospitalized refracture and competing death among patients with a survival time of ≥30 days after discharge. We additionally compared cause-specific and subdistribution hazard estimates and quantified clinically meaningful state occupation probabilities using a multistate framework.

## Materials and methods

### Data source and study design

This study was analyzed based on a de-identified public longitudinal dataset originally described in a Scientific Data data descriptor (11). The dataset was derived from the Osteoporotic Fracture Registration System at the Affiliated Kunshan Hospital of Jiangsu University and linked with regional health and mortality registries. The source descriptor reports that the Clinical Variables Table includes 4,782 registered patients and that the Medication Table includes 42,367 longitudinal antihypertensive prescription records. The current study focused on competing-risk and multistate characterization of refracture and death after osteoporotic fracture.

### Study population

The source population included adults aged 50 years or older who were hospitalized for osteoporotic fracture between January 1, 2017, and April 24, 2024. Index sites included wrist/radius, proximal humerus, thoracic vertebra, lumbar vertebra, and hip fractures. The primary long-term cohort was defined as patients with Survivaltime ≥30 days and available event variables. Thirty-one patients with Survivaltime <30 days were excluded; all died within 0–26 days after discharge. Of these early deaths, 27 followed hip fracture, 3 lumbar vertebral fracture, and 1 thoracic vertebral fracture. One patient who died exactly on day 30 remained in the cohort. This yielded 4,751 patients for the primary analysis.

The 30-day-survivor restriction was retained because the prespecified objective concerned long-term trajectories. This landmark-type restriction conditions all inference on survival to the 30-day time point (13). Excluding the 31 early deaths, most of whom had hip fracture, removes patients at the highest acute mortality risk and may reduce the apparent competing burden of death relative to the full source cohort. The public Deathreason variable classified 16 early deaths as fall-related and 15 as non-fall-related but did not identify pulmonary embolism, perioperative complications, or other specific causes; these mechanisms therefore could not be evaluated.

### Outcomes and competing-risk structure

The primary outcome was first recorded hospitalized refracture after the index osteoporotic fracture, identified from Refracture, Refracture1Date, and Refracturetime. This terminology reflects the hospitalization-based ascertainment available in the public dataset and does not imply complete capture of asymptomatic or mildly symptomatic vertebral fractures, outpatient-managed fractures, out-of-region events, or events not brought to medical attention (14, 15). The competing event was all-cause death before the target event, identified from Death_all, Deathtime, and Survivaltime.

The four-state model comprised alive without recorded refracture, alive after recorded refracture, death without recorded refracture, and death after recorded refracture. The primary multistate analysis used the common refracture observation horizon of March 24, 2024; the complete mortality window through April 24, 2024 was evaluated in sensitivity analysis. Follow-up was calculated from discharge after the index hospitalization. Transition estimates involving death after refracture were reported descriptively because of the limited number of observed transitions.

### Covariates

Candidate covariates were selected *a priori* according to clinical relevance, availability, and missingness. Core covariates included age, sex, body mass index (BMI), fracture site, Charlson Comorbidity Index (CCI) (16), recorded hypertension history, Fracture Liaison Service (FLS) status (17, 18), and recorded discharge care-pathway indicators. Fracture site was categorized as wrist/radius, proximal humerus, thoracic vertebra, lumbar vertebra, or hip, with wrist/radius as the regression reference. Age was modeled per 10-year increment, and CCI was categorized as 0, 1, or ≥2.

Recorded discharge care-pathway covariates included osteoporosis-related medication indicators. These variables were used only for adjustment and descriptive association, not causal treatment inference. No treatment effect was inferred from these associations.

### Missing data

Missingness was evaluated before model construction. Variables with high missingness were excluded from primary regression models *a priori*. Bone mineral density variables, 25-hydroxyvitamin D, C-reactive protein, beta-C-terminal telopeptide, procollagen type I N-terminal propeptide, and parathyroid hormone had substantial missingness and were not included in the primary models. These variables were considered unsuitable as main exposures or mandatory covariates in the full cohort analysis and were reserved for exploratory or supplementary analyses only.

Complete-case regression was used for multivariable cause-specific Cox and Fine–Gray models because high-missingness variables were excluded *a priori* and the primary objective was estimation of association patterns rather than individualized prediction. Because complete-case estimates may be selected when missingness is related to analysis variables, this choice was treated as a potential source of selection bias (19). The complete-case regression cohort contained 4,001 patients. Missing data patterns were summarized in **Supplementary Table 1** and **Supplementary Figure 1**.

### Statistical analysis

Baseline characteristics were summarized by corrected first-event status. Cumulative incidence functions were estimated nonparametrically for first recorded hospitalized refracture and death before recorded refracture at 1, 3, and 5 years. Death was treated as a competing event when estimating refracture occurrence. Estimates were accompanied by 95% confidence intervals, and risk-set, event, and censoring counts were audited.

Cause-specific Cox models estimated associations with the instantaneous hazard of first recorded hospitalized refracture among patients remaining alive and refracture-free. Fine–Gray models estimated associations with the refracture cumulative incidence function in the presence of competing death (6–8). Identical complete-case data and covariates were used in both models. Results are reported as hazard ratios (HRs) and subdistribution hazard ratios (sHRs), which represent distinct estimands. Cox proportional hazards assumptions were assessed with Schoenfeld residuals.

Aalen–Johansen estimates were used for 1-, 3-, and 5-year state-occupation probabilities (8). All-cause mortality was additionally evaluated using a prespecified Cox model. The primary common-horizon multistate analysis was supplemented by a full mortality-window analysis. The four state probabilities were verified to sum to 1 within numerical precision at each reported time point. Analyses were performed in R using the survival and cmprsk packages, with code retained for reproducibility.

Prespecified sensitivity analyses evaluated Survivaltime >30 days, exclusion of the time-zero refracture, the submitted strict administrative-boundary rule, death-first assignment for the same-day refracture/death record, inclusion of all 4,782 source patients, exclusion of first events within 90 days, and use of the full mortality window. Index-fracture heterogeneity was evaluated descriptively using site-specific patient characteristics, first-event counts, and 1-, 3-, and 5-year cumulative incidence; no underpowered site-specific multivariable interaction analysis was undertaken.

## Results

### Study cohort and event distribution

4,782 patients were included. In accordance with the prespecified 30-day survivor criterion, 31 patients who died within 0–26 days after discharge were excluded, leaving 4,751 patients in the primary cohort (**Figure 1**; **Table 1**). Baseline characteristics according to first-event status are summarized in **Table 1**. The complete-case cohort used for the cause-specific Cox and Fine–Gray analyses comprised 4,001 patients, including 212 patients with a first recorded hospitalized refracture.

**Figure 1:**
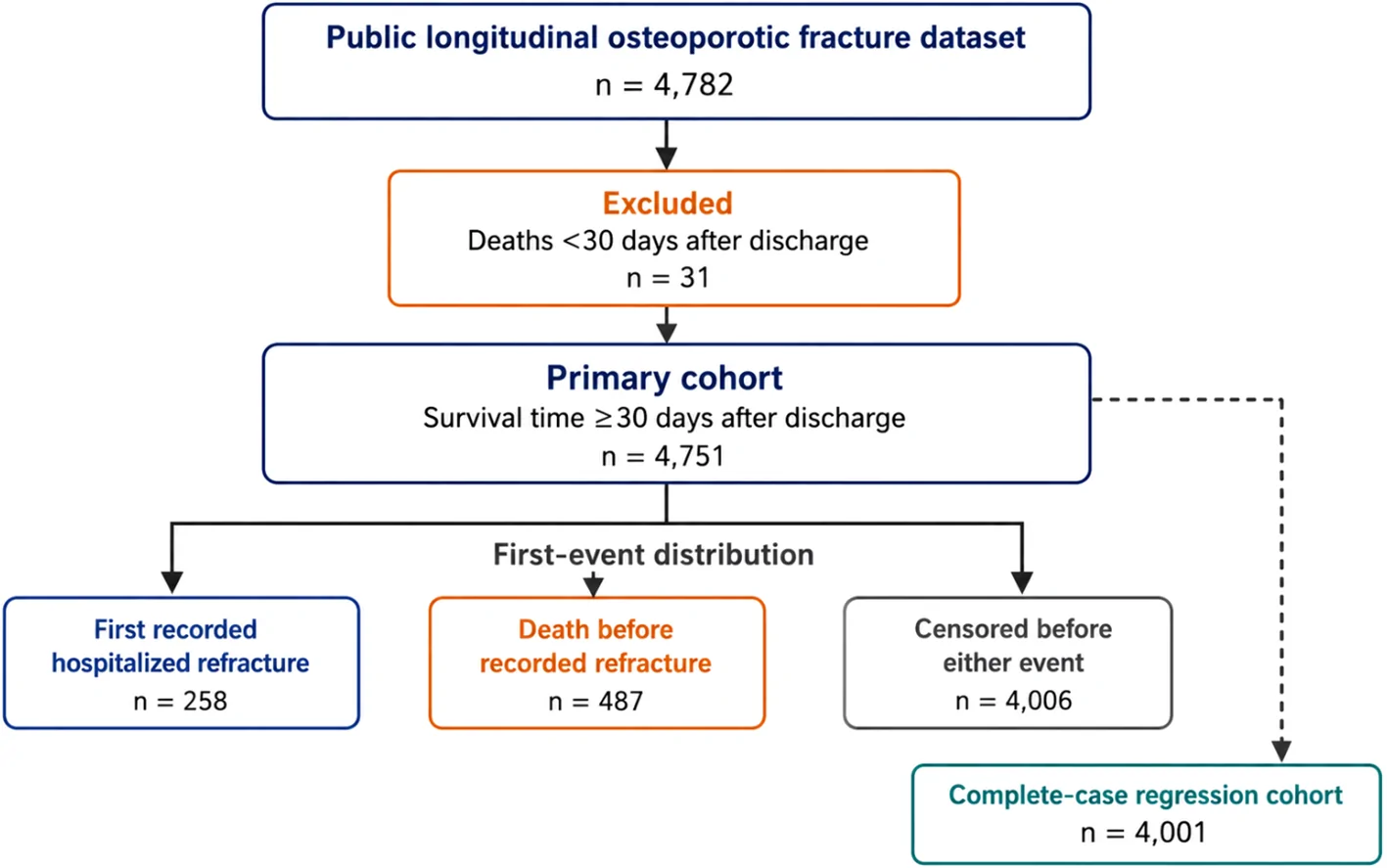
Study flow diagram. The source cohort contained 4,782 patients. Thirty-one patients died within 0–26 days and were excluded by the prespecified 30-day-survivor definition, leaving 4,751 patients. Corrected first-event categories comprised 258 first recorded hospitalized refractures, 487 deaths before recorded refracture, and 4,006 censored observations. The complete-case regression cohort contained 4,001 patients and 212 target events.

**Table 1:** Baseline characteristics by first event status.

Variable	Overall	Censored before either event	First recorded hospitalized refracture	Death before recorded refracture
Age, years	68.7 (11.2)	66.9 (10.5)	72.3 (10.3)	81.3 (8.0)
BMI, kg/m²	23.3 (3.3)	23.4 (3.2)	23.0 (3.3)	22.0 (3.5)
Male sex	1528 (32.2)	1284 (32.1)	57 (22.1)	187 (38.4)
Index fracture site				
Wrist/radius	668 (14.1)	627 (15.7)	27 (10.5)	14 (2.9)
Proximal humerus	410 (8.6)	392 (9.8)	11 (4.3)	7 (1.4)
Thoracic vertebra	722 (15.2)	636 (15.9)	49 (19.0)	37 (7.6)
Lumbar vertebra	1287 (27.1)	1128 (28.2)	81 (31.4)	78 (16.0)
Hip fracture	1664 (35.0)	1223 (30.5)	90 (34.9)	351 (72.1)
CCI category				
0	4244 (89.3)	3631 (90.6)	225 (87.2)	388 (79.7)
1	394 (8.3)	297 (7.4)	22 (8.5)	75 (15.4)
≥2	113 (2.4)	78 (1.9)	11 (4.3)	24 (4.9)
Recorded FLS use	1291 (27.2)	1093 (27.3)	62 (24.0)	136 (27.9)
Recorded hypertension history	650 (13.7)	464 (11.6)	43 (16.7)	143 (29.4)
Calcium indicator	2060 (43.4)	1733 (43.3)	99 (38.4)	228 (46.8)
Alfacalcidol indicator	1974 (41.5)	1560 (38.9)	139 (53.9)	275 (56.5)
Vitamin D2 indicator	1106 (23.3)	1030 (25.7)	16 (6.2)	60 (12.3)
Zoledronic acid indicator	1225 (25.8)	1035 (25.8)	57 (22.1)	133 (27.3)
Calcitonin indicator	1087 (22.9)	964 (24.1)	38 (14.7)	85 (17.5)
Alendronate indicator	91 (1.9)	56 (1.4)	14 (5.4)	21 (4.3)

Values are mean (SD) or n (%); valid N is 4,001 for BMI and 4,751 for variables without missingness. Event groups contain 4,006 censored patients, 258 patients with first recorded hospitalized refracture, and 487 patients with death before recorded refracture. BMI, body mass index; CCI, Charlson Comorbidity Index; FLS, Fracture Liaison Service.

During follow-up, 258 patients experienced a first recorded hospitalized refracture as their first event, 487 died before a recorded refracture, and 4,006 were censored before either event. In the primary analysis, an observed death occurring on the administrative censoring boundary was classified as a competing event. Alternative classification of this record was examined in a sensitivity analysis.

### Cumulative incidence of refracture and competing death

At 5 years after discharge, the cumulative incidence of first recorded hospitalized refracture was 8.63% (95% CI, 7.51–9.75%), whereas that of death before recorded refracture was 15.57% (95% CI, 14.12–17.03%) (**Figure 2**; **Table 2**). Thus, competing death was more frequent than recorded refracture over the 5-year follow-up period. Under the alternative administrative-boundary rule, the corresponding estimates were 8.63% and 15.54%, respectively.

**Figure 2:**
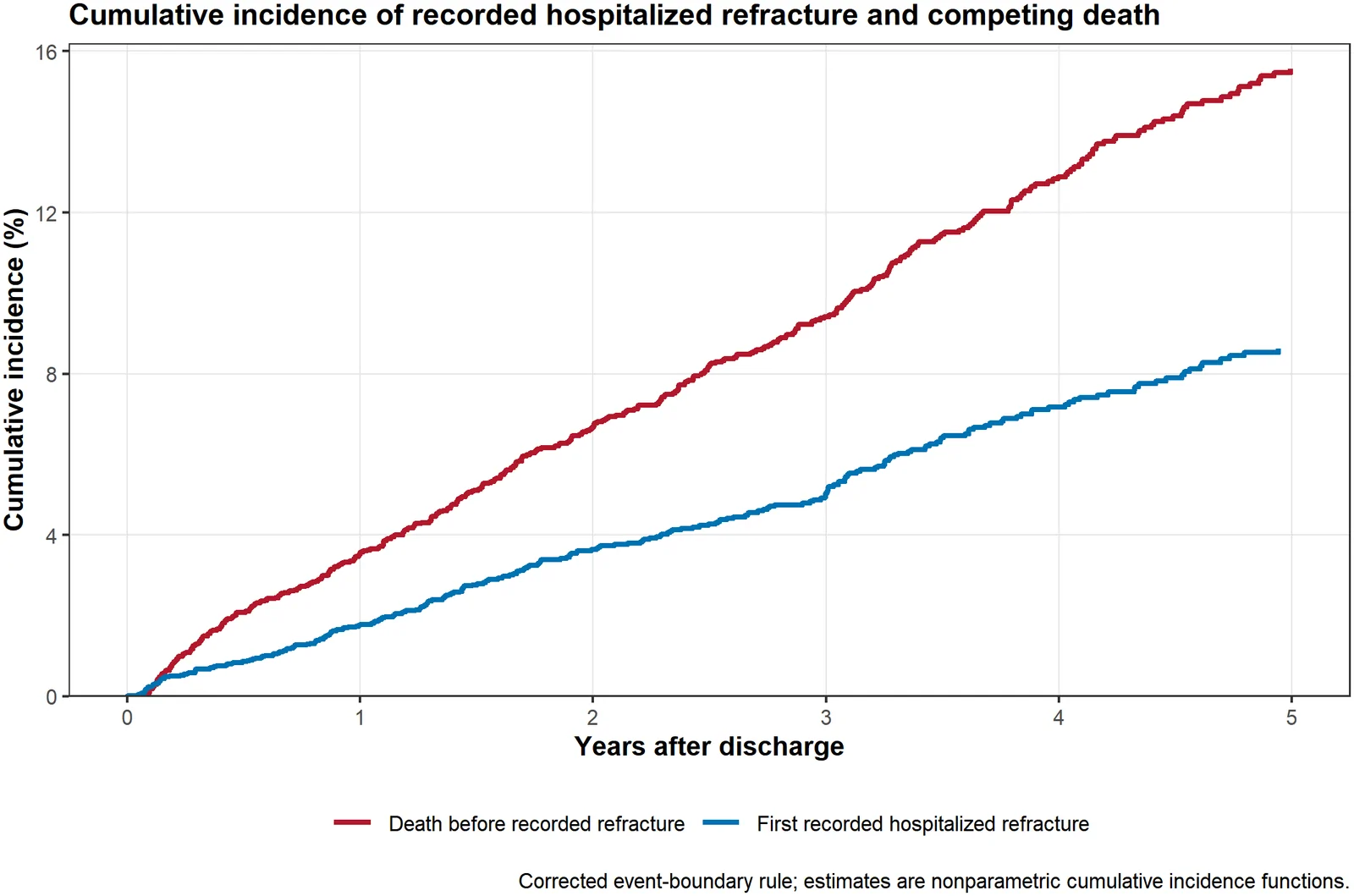
Cumulative incidence functions for first recorded hospitalized refracture and death before recorded refracture.

**Table 2:** Cumulative incidence and multistate probabilities at 1, 3, and 5 years.

Time, years	First recorded hospitalized refracture CIF, %	Death before recorded refracture CIF, %	Alive without recorded refracture, %	Alive after recorded refracture, %	Death without recorded refracture, %	Death after recorded refracture, %
1.00	1.79	3.58	94.63	1.72	3.58	0.07
3.00	5.04	9.42	85.54	4.32	9.42	0.72
5.00	8.63	15.57	75.80	6.76	15.57	1.88

CIF, cumulative incidence function. Death before recorded refracture was treated as a competing event. State-occupation probabilities were estimated using the Aalen-Johansen method under the common administrative horizon and sum to 100% apart from rounding. Refracture refers to first recorded hospitalized refracture.

### Factors associated with refracture and mortality

In the complete-case analyses, older age, thoracic or lumbar vertebral fracture rather than wrist/radius fracture, and a Charlson Comorbidity Index (CCI) of 2 or higher were associated with higher refracture estimates, whereas male sex was associated with lower estimates. The directions of association were generally consistent between the cause-specific Cox and Fine–Gray models, and classification of the additional competing death did not change the statistical-significance status of any covariate (**Figure 3**; **Supplementary Table 3**). Recorded discharge care-pathway covariates showed heterogeneous associations across the two models.

**Figure 3:**
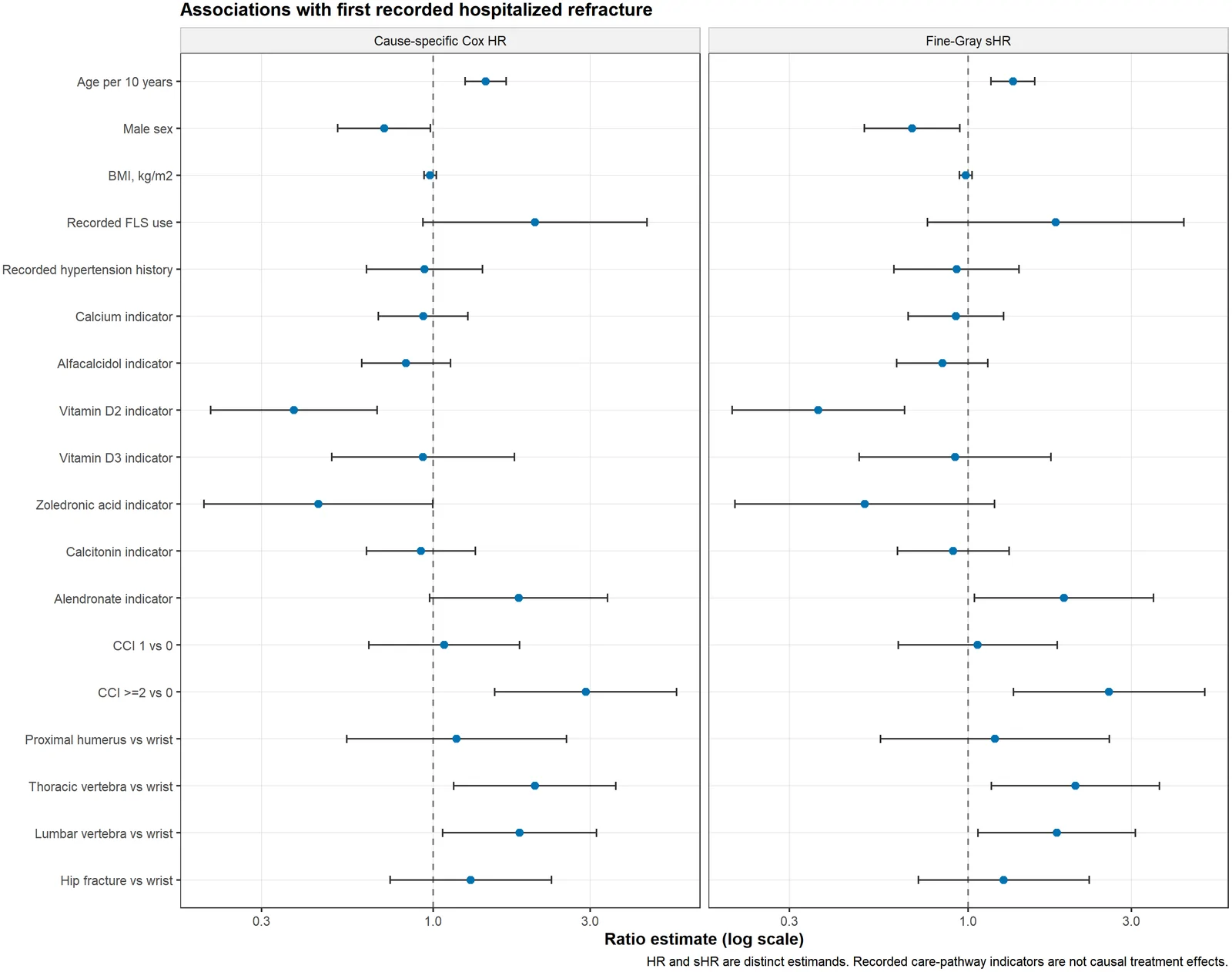
Cause-specific Cox and fine-gray estimates for first recorded hospitalized refracture. Cause-specific hazard ratios (HRs), and Fine-Gray subdistribution hazard ratios (sHRs) are distinct estimands. BMI, body mass index; CCI, Charlson Comorbidity Index; FLS, Fracture Liaison Service.

The all-cause mortality analysis included 4,001 complete cases and 373 deaths. Older age, male sex, lower body mass index, greater comorbidity burden, and hip fracture rather than wrist/radius fracture were associated with higher mortality estimates (**Supplementary Table 4**). The global test provided no evidence of violation of the proportional hazards assumption (p = 0.216).

### Multistate trajectories

At 5 years, the estimated Aalen–Johansen state probabilities were 75.80% for being alive without a recorded refracture, 6.76% for being alive after a recorded refracture, 15.57% for death without a recorded refracture, and 1.88% for death after a recorded refracture (**Figure 4**; **Table 2**).

**Figure 4:**
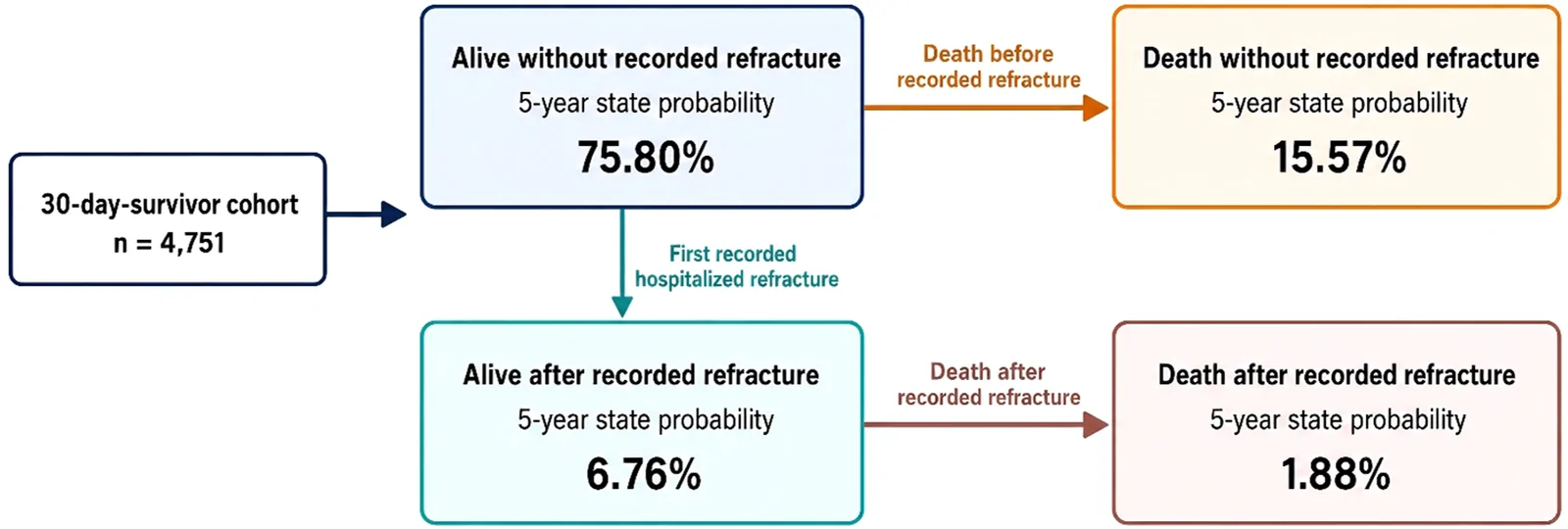
Multistate transitions after index osteoporotic fracture. Corrected 5-year Aalen-Johansen state probabilities were 75.80% alive without recorded refracture, 6.76% alive after recorded refracture, 15.57% dead without recorded refracture, and 1.88% dead after recorded refracture. The common-horizon model included 52 refracture-to-death transitions; 53 such sequences were observed during full mortality follow-up, including 47 by 5 years.

The multistate dataset included 52 observed transitions from recorded refracture to death over the analysis horizon. Across the extended mortality follow-up, 53 patients died after a recorded refracture, including 47 deaths within 5 years (**Supplementary Table 5**). Estimates for the death-after-refracture state were therefore based on relatively few events and had lower precision than estimates for the other states.

### Sensitivity and fracture-site analyses

The sensitivity analyses yielded results consistent with the primary findings. When all 4,782 source patients were included, the 5-year cumulative incidence of competing death increased from 15.57% to 16.10%, consistent with the exclusion of early deaths from the 30-day survivor cohort. Alternative handling of the administrative-boundary record, exclusion of the time-zero event, and reversal of the ordering of same-day events produced only minor numerical changes.

Descriptive fracture-site analyses showed substantial clinical heterogeneity. Patients with hip fracture were older than those in the other fracture groups (mean age, 74.8 years) and had a 5-year competing-death cumulative incidence of 29.83%, compared with 3.45% among patients with wrist/radius fracture. The 5-year cumulative incidence of recorded refracture was 10.20% after thoracic vertebral fracture, 10.03% after lumbar vertebral fracture, 7.66% after hip fracture, and 7.11% after wrist/radius fracture (**Supplementary Table 2**).

## Discussion

Among patients who survived for at least 30 days after discharge following an osteoporotic fracture, the 5-year cumulative incidence of death before recorded refracture was 15.57%, compared with 8.63% for first recorded hospitalized refracture. The multistate analysis similarly showed that death without a prior recorded refracture was substantially more common than death after refracture. These findings indicate that mortality materially shapes the observable long-term course after osteoporotic fracture and should be treated as a competing event when estimating the absolute occurrence of refracture.

This distinction is methodologically important. When death is treated as ordinary censoring in Kaplan–Meier estimation, the resulting estimate may overstate the cumulative probability of refracture because patients who die can no longer experience a subsequently recorded refracture. The cumulative incidence function addresses this issue by estimating refracture occurrence in the presence of competing death. Nevertheless, the present estimate represents the occurrence of recorded hospitalized refractures rather than that of all clinical or radiographic fractures.

Cause-specific Cox and Fine–Gray models address related but distinct questions and are not interchangeable (6–8). The cause-specific model evaluates associations with the instantaneous rate of refracture among patients who remain alive and refracture-free, whereas the Fine–Gray model evaluates associations with the cumulative incidence of refracture while retaining competing deaths in the risk set. Reporting both models therefore provides complementary perspectives on event-specific and cumulative-incidence associations. Neither model, however, establishes causality when applied to observational data.

The multistate framework extends these analyses by distinguishing four clinically meaningful states: alive without recorded refracture, alive after recorded refracture, death without recorded refracture, and death after recorded refracture. This framework illustrates how patients move between clinically meaningful states rather than reducing follow-up to a single endpoint. Within the common administrative follow-up horizon used for the primary Aalen–Johansen analysis, 52 transitions from recorded refracture to death were observed. Across the extended mortality follow-up, 53 patients died after a recorded refracture, including 47 deaths within 5 years. Because these events represented only a small proportion of the cohort, the 1.88% probability of death after refracture and the corresponding transition-specific estimates should be considered descriptive. Larger datasets with more complete fracture and mortality histories will be required for precise modeling of this transition.

The fracture-site analyses further demonstrated substantial clinical heterogeneity. Patients with hip fracture were older and had a markedly higher 5-year cumulative incidence of competing death, whereas patients with thoracic or lumbar vertebral fractures had higher cumulative incidences of recorded refracture. These findings are consistent with previous evidence that prognosis differs considerably across osteoporotic fracture types (4, 5). Index fracture site should therefore be regarded as a marker of a broader clinical profile rather than an isolated determinant of outcome. For example, prognosis in a relatively young patient with a wrist fracture cannot be directly extrapolated to that of a very old patient with a hip fracture, because the two patients may also differ in frailty, comorbidity, functional status, treatment requirements, and baseline mortality risk. Multivariable adjustment can reduce measured confounding but cannot fully account for this heterogeneity. The site-specific analyses were descriptive and require confirmation through adequately powered stratified or interaction analyses in larger external cohorts.

The present findings are consistent with the broader literature showing that patients with osteoporotic fractures face substantial risks of both subsequent fracture and death. Previous studies have explicitly incorporated death as a competing event when estimating post-fracture probabilities (3). The AGES–Reykjavik study used multistate methods to examine refracture and mortality among older adults with osteoporotic fractures (9), and more recent fracture-risk research has emphasized the importance of accounting for competing mortality when estimating long-term fracture probability (10). The present study extends this work by applying complementary competing-risk and multistate methods to a Chinese longitudinal cohort.

These findings do not diminish the importance of secondary fracture prevention. Rather, they suggest that refracture prevention should be considered within the broader clinical trajectory of older fracture survivors. Post-fracture care may therefore benefit from integrating osteoporosis treatment with comorbidity management, rehabilitation, fall prevention, and assessment of mortality and functional risk (1, 2, 17, 18). Such an integrated approach may be particularly important for older patients with hip fracture or a substantial comorbidity burden.

Associations involving recorded discharge care-pathway covariates require particular caution. These variables may reflect fracture severity, clinician selection, access to care, patient prognosis, adherence potential, and other health-system factors. Consequently, the observed associations do not provide evidence that a treatment or care pathway was beneficial or harmful. Evaluating the comparative effectiveness of osteoporosis treatments or post-fracture care pathways would require a dedicated causal-inference design with more comprehensive adjustment for confounding by indication.

An additional contribution of this study is that it addresses a question distinct from the hypertension-centered technical validation reported in the original data descriptor (11). Whereas the original publication demonstrated the structure and utility of the dataset through hypertension-related analyses, the present study focused specifically on the competing and sequential occurrence of refracture and death.

This study has several strengths. Competing-risk regression and multistate estimation were applied to provide complementary descriptions of post-fracture outcomes. Sensitivity analyses examining the 30-day survivor restriction, administrative-boundary classification, time-zero events, and same-day event ordering produced only minor numerical changes. In addition, variables with extensive missingness were not forced into the primary models, reducing the risk of unstable estimates arising from sparse data.

Several limitations should be considered. First, this was a secondary analysis of observational data and therefore remains susceptible to residual and unmeasured confounding. Restricting the primary cohort to 30-day survivors introduced survivorship bias and limits the generalizability of the findings to patients who survived the early post-discharge period (13). Most of the 31 excluded early deaths occurred after hip fracture, consistent with the high early mortality and perioperative risk reported in hip-fracture populations (5, 20). However, the dataset did not provide sufficient information to determine whether these deaths resulted from pulmonary embolism, surgical complications, infection, underlying frailty, or other causes. Excluding these patients reduced the observed burden of competing death, as supported by the higher 5-year death incidence when the entire source cohort was analyzed.

Second, follow-up began at discharge rather than at the date of fracture or hospital admission. The estimates may therefore not be directly comparable with studies using fracture occurrence or admission as time zero. More importantly, refracture ascertainment relied on hospitalization records. Asymptomatic or mildly symptomatic vertebral fractures, fractures managed exclusively in outpatient settings, events occurring outside the participating health system, and fractures for which medical attention was not sought may have been missed (14, 15). This incomplete capture probably led to underestimation of refracture incidence and may have made death appear relatively more prominent as a competing event.

Third, pooling different index fracture sites may obscure clinically meaningful differences in age, frailty, management, and prognosis. Although fracture site and other measured characteristics were included in the regression models, adjustment cannot eliminate all residual heterogeneity. The fracture-site analyses were descriptive because some strata contained limited numbers of events, and the findings require confirmation in larger site-specific cohorts.

Finally, information on fracture severity, frailty, physical function, falls, treatment adherence, socioeconomic circumstances, and several other potential confounders was unavailable. Bone-health and inflammatory biomarkers also had substantial missingness and could not be incorporated reliably into the primary models. Complete-case regression may have introduced additional selection bias if the included patients differed systematically from those with missing covariate information (19). Furthermore, the limited number of refracture-to-death transitions reduced the precision of estimates for the death-after-refracture state.

Overall, death was a prominent competing event during the 5 years following osteoporotic fracture and occurred more frequently than first recorded hospitalized refracture among 30-day survivors. Competing-risk and multistate analyses provided complementary estimates of event occurrence and post-fracture state occupancy. These estimates were conditional on survival beyond 30 days and were limited by incomplete ascertainment of non-hospitalized fractures, heterogeneity across fracture sites, and the small number of deaths following refracture.

## Conclusion

Among patients who survived for at least 30 days after discharge following an osteoporotic fracture, death before recorded refracture was more common than first recorded hospitalized refracture over 5 years. Competing-risk and multistate analyses quantified cumulative event occurrence and post-fracture state occupancy, respectively. These findings apply to 30-day survivors and to refractures captured through hospitalization records, with prognosis varying substantially across index fracture sites.

## Data Availability

The original contributions presented in the study are included in the article/**Supplementary Material**. Further inquiries can be directed to the corresponding authors.
