# Supplementary material for: Refracture or death after osteoporotic fracture: five-year competing-risk and multistate trajectories
Source: Front Endocrinol (Lausanne). 2026 Sep 16;17:1905993. doi: 10.3389/fendo.2026.1905993 (PMC13629301; doi:10.3389/fendo.2026.1905993)
Supplement: Supplementary file 1 [file SupplementaryFile1.docx]

**Supplementary Figure S1. Missingness map.**

Missingness map. The stacked bar map summarizes missingness in selected candidate variables. High-missingness variables, including bone mineral density variables, 25-hydroxyvitamin D, C-reactive protein, beta-C-terminal telopeptide, procollagen type I N-terminal propeptide, and parathyroid hormone, were excluded from the primary regression models.

**Supplementary Table S1. Missingness of candidate variables in the primary cohort**

| **Variable** | **Missing, n** | **Missing, %** | **Nonmissing, n** |
| --- | --- | --- | --- |
| 25-hydroxyvitamin D | 4,183 | 88.04 | 568 |
| C-reactive protein | 4,085 | 85.98 | 666 |
| Beta-C-terminal telopeptide | 3,665 | 77.14 | 1,086 |
| Procollagen type 1 N-terminal propeptide | 3,664 | 77.12 | 1,087 |
| Femoral neck BMD | 3,097 | 65.19 | 1,654 |
| Intertrochanteric BMD | 3,097 | 65.19 | 1,654 |
| Total hip BMD | 3,097 | 65.19 | 1,654 |
| Trochanter BMD | 3,097 | 65.19 | 1,654 |
| Ward's triangle BMD | 3,097 | 65.19 | 1,654 |
| Lumbar BMD | 3,073 | 64.68 | 1,678 |
| Parathyroid hormone | 2,404 | 50.60 | 2,347 |
| Body mass index | 750 | 15.79 | 4,001 |
| Systolic blood pressure | 658 | 13.85 | 4,093 |
| Diastolic blood pressure | 657 | 13.83 | 4,094 |
| Hemoglobin | 88 | 1.85 | 4,663 |
| Age | 0 | 0.00 | 4,751 |
| Alendronate indicator | 0 | 0.00 | 4,751 |
| Alfacalcidol indicator | 0 | 0.00 | 4,751 |
| Calcitonin indicator | 0 | 0.00 | 4,751 |
| Calcium indicator | 0 | 0.00 | 4,751 |
| Charlson Comorbidity Index | 0 | 0.00 | 4,751 |
| Discharge date | 0 | 0.00 | 4,751 |
| Fracture Liaison Service | 0 | 0.00 | 4,751 |
| Index fracture site | 0 | 0.00 | 4,751 |
| Recorded hypertension history | 0 | 0.00 | 4,751 |
| Sex | 0 | 0.00 | 4,751 |
| Vitamin D2 indicator | 0 | 0.00 | 4,751 |
| Vitamin D3 indicator | 0 | 0.00 | 4,751 |
| Zoledronic acid indicator | 0 | 0.00 | 4,751 |

Note. The primary cohort included 4,751 patients with Survivaltime >=30 days. Mixed-format dates were normalized before missingness was assessed; DischargeDate had no missing observations. BMD, bone mineral density; 25VD, 25-hydroxyvitamin D; CRP, C-reactive protein; BTX, beta-C-terminal telopeptide; P1NP, procollagen type 1 N-terminal propeptide; PTH, parathyroid hormone.

**Supplementary Table S2. Prespecified sensitivity analyses for first recorded refracture and competing death**

| **Analysis** | **Cohort or event rule** | **Year** | **N** | **First refractures, n** | **Competing deaths, n** | **Censored, n** | **Refracture CIF (95% CI)** | **Competing-death CIF (95% CI)** |
| --- | --- | --- | --- | --- | --- | --- | --- | --- |
| Primary corrected rule: Survivaltime >=30 | Primary comparator | 1 | 4,751 | 258 | 487 | 4,006 | 1.79% (1.41-2.17) | 3.58% (3.05-4.12) |
| Primary corrected rule: Survivaltime >=30 | Primary comparator | 3 | 4,751 | 258 | 487 | 4,006 | 5.04% (4.32-5.75) | 9.42% (8.47-10.38) |
| Primary corrected rule: Survivaltime >=30 | Primary comparator | 5 | 4,751 | 258 | 487 | 4,006 | 8.63% (7.51-9.75) | 15.57% (14.12-17.03) |
| Survivaltime >30 | Exclude the day-30 death | 1 | 4,750 | 258 | 486 | 4,006 | 1.79% (1.41-2.17) | 3.56% (3.03-4.10) |
| Survivaltime >30 | Exclude the day-30 death | 3 | 4,750 | 258 | 486 | 4,006 | 5.04% (4.32-5.76) | 9.40% (8.45-10.36) |
| Survivaltime >30 | Exclude the day-30 death | 5 | 4,750 | 258 | 486 | 4,006 | 8.63% (7.51-9.75) | 15.55% (14.10-17.01) |
| Exclude primary-cohort time-zero refracture | Remove the source-recorded time-zero refracture | 1 | 4,750 | 257 | 487 | 4,006 | 1.77% (1.39-2.15) | 3.58% (3.05-4.12) |
| Exclude primary-cohort time-zero refracture | Remove the source-recorded time-zero refracture | 3 | 4,750 | 257 | 487 | 4,006 | 5.02% (4.30-5.73) | 9.42% (8.47-10.38) |
| Exclude primary-cohort time-zero refracture | Remove the source-recorded time-zero refracture | 5 | 4,750 | 257 | 487 | 4,006 | 8.61% (7.49-9.73) | 15.58% (14.12-17.03) |
| Strict death-before-administrative-boundary rule | Use the submitted strict < boundary rule | 1 | 4,751 | 258 | 486 | 4,007 | 1.79% (1.41-2.17) | 3.58% (3.05-4.12) |
| Strict death-before-administrative-boundary rule | Use the submitted strict < boundary rule | 3 | 4,751 | 258 | 486 | 4,007 | 5.04% (4.32-5.75) | 9.39% (8.43-10.34) |
| Strict death-before-administrative-boundary rule | Use the submitted strict < boundary rule | 5 | 4,751 | 258 | 486 | 4,007 | 8.63% (7.51-9.75) | 15.54% (14.09-17.00) |
| Same-day refracture/death assigned death first | Assign the same-day record to death first | 1 | 4,751 | 257 | 488 | 4,006 | 1.79% (1.41-2.17) | 3.58% (3.05-4.12) |
| Same-day refracture/death assigned death first | Assign the same-day record to death first | 3 | 4,751 | 257 | 488 | 4,006 | 5.04% (4.32-5.75) | 9.42% (8.47-10.38) |
| Same-day refracture/death assigned death first | Assign the same-day record to death first | 5 | 4,751 | 257 | 488 | 4,006 | 8.59% (7.47-9.70) | 15.62% (14.16-17.08) |
| Full source cohort including early deaths | Include all 4,782 source patients | 1 | 4,782 | 259 | 517 | 4,006 | 1.80% (1.42-2.18) | 4.19% (3.61-4.76) |
| Full source cohort including early deaths | Include all 4,782 source patients | 3 | 4,782 | 259 | 517 | 4,006 | 5.03% (4.32-5.74) | 9.99% (9.02-10.96) |
| Full source cohort including early deaths | Include all 4,782 source patients | 5 | 4,782 | 259 | 517 | 4,006 | 8.60% (7.48-9.71) | 16.10% (14.64-17.56) |
| Event-free at 90 days; time reset at day 90 | Exclude first events during the first 90 days | 1 | 4,674 | 232 | 436 | 4,006 | 1.63% (1.25-2.00) | 3.26% (2.74-3.78) |
| Event-free at 90 days; time reset at day 90 | Exclude first events during the first 90 days | 3 | 4,674 | 232 | 436 | 4,006 | 5.25% (4.48-6.02) | 9.53% (8.52-10.54) |
| Event-free at 90 days; time reset at day 90 | Exclude first events during the first 90 days | 5 | 4,674 | 232 | 436 | 4,006 | 8.42% (7.27-9.57) | 15.28% (13.75-16.80) |
| Index fracture site: Wrist/radius | Descriptive site-specific competing-risk analysis | 1 | 668 | 27 | 14 | 627 | 1.08% (0.28-1.88) | 0.79% (0.10-1.49) |
| Index fracture site: Wrist/radius | Descriptive site-specific competing-risk analysis | 3 | 668 | 27 | 14 | 627 | 4.54% (2.59-6.50) | 1.81% (0.60-3.01) |
| Index fracture site: Wrist/radius | Descriptive site-specific competing-risk analysis | 5 | 668 | 27 | 14 | 627 | 7.11% (4.27-9.94) | 3.45% (1.37-5.53) |
| Index fracture site: Proximal humerus | Descriptive site-specific competing-risk analysis | 1 | 410 | 11 | 7 | 392 | 0.53% (0.00-1.27) | 0.49% (0.00-1.16) |
| Index fracture site: Proximal humerus | Descriptive site-specific competing-risk analysis | 3 | 410 | 11 | 7 | 392 | 4.81% (1.24-8.38) | 3.02% (0.28-5.76) |
| Index fracture site: Proximal humerus | Descriptive site-specific competing-risk analysis | 5 | 410 | 11 | 7 | 392 | 8.84% (3.18-14.50) | 4.10% (0.66-7.54) |
| Index fracture site: Thoracic vertebra | Descriptive site-specific competing-risk analysis | 1 | 722 | 49 | 37 | 636 | 1.99% (0.96-3.03) | 1.71% (0.75-2.66) |
| Index fracture site: Thoracic vertebra | Descriptive site-specific competing-risk analysis | 3 | 722 | 49 | 37 | 636 | 6.40% (4.35-8.45) | 4.20% (2.50-5.89) |
| Index fracture site: Thoracic vertebra | Descriptive site-specific competing-risk analysis | 5 | 722 | 49 | 37 | 636 | 10.20% (7.30-13.11) | 8.23% (5.35-11.12) |
| Index fracture site: Lumbar vertebra | Descriptive site-specific competing-risk analysis | 1 | 1,287 | 81 | 78 | 1,128 | 2.22% (1.40-3.03) | 1.82% (1.08-2.55) |
| Index fracture site: Lumbar vertebra | Descriptive site-specific competing-risk analysis | 3 | 1,287 | 81 | 78 | 1,128 | 5.39% (4.03-6.75) | 4.98% (3.67-6.30) |
| Index fracture site: Lumbar vertebra | Descriptive site-specific competing-risk analysis | 5 | 1,287 | 81 | 78 | 1,128 | 10.03% (7.66-12.40) | 8.61% (6.48-10.74) |
| Index fracture site: Hip | Descriptive site-specific competing-risk analysis | 1 | 1,664 | 90 | 351 | 1,223 | 1.94% (1.28-2.61) | 7.59% (6.31-8.88) |
| Index fracture site: Hip | Descriptive site-specific competing-risk analysis | 3 | 1,664 | 90 | 351 | 1,223 | 4.61% (3.51-5.72) | 18.98% (16.87-21.09) |
| Index fracture site: Hip | Descriptive site-specific competing-risk analysis | 5 | 1,664 | 90 | 351 | 1,223 | 7.66% (5.99-9.32) | 29.83% (26.85-32.82) |

Note. CIF, cumulative incidence function; CI, confidence interval. The target outcome is first recorded hospitalized refracture. Estimates were generated at 1, 3, and 5 years under each prespecified rule and for each index fracture site. Site-specific estimates are descriptive and were not used for post hoc interaction selection. The primary corrected rule prioritizes an observed death over same-day administrative censoring.

**Supplementary Table S3. Cause-specific Cox and Fine-Gray models for first recorded refracture**

| **Variable** | **Cause-specific HR (95% CI)** | **P value** | **Fine-Gray sHR (95% CI)** | **P value** | **Model N** | **First-refracture events, n** |
| --- | --- | --- | --- | --- | --- | --- |
| Age per 10 years | 1.44 (1.25-1.67) | <0.001 | 1.35 (1.17-1.57) | <0.001 | 4,001 | 212 |
| Male sex | 0.71 (0.51-0.98) | 0.038 | 0.69 (0.50-0.95) | 0.021 | 4,001 | 212 |
| BMI, kg/m2 | 0.98 (0.94-1.02) | 0.311 | 0.98 (0.94-1.02) | 0.407 | 4,001 | 212 |
| Recorded FLS use | 2.04 (0.93-4.48) | 0.075 | 1.80 (0.76-4.27) | 0.181 | 4,001 | 212 |
| Recorded hypertension history | 0.94 (0.63-1.41) | 0.767 | 0.92 (0.61-1.41) | 0.714 | 4,001 | 212 |
| Calcium indicator | 0.93 (0.68-1.28) | 0.659 | 0.92 (0.67-1.27) | 0.606 | 4,001 | 212 |
| Alfacalcidol indicator | 0.83 (0.61-1.13) | 0.228 | 0.84 (0.62-1.14) | 0.267 | 4,001 | 212 |
| Vitamin D2 indicator | 0.38 (0.21-0.67) | 0.001 | 0.36 (0.20-0.65) | <0.001 | 4,001 | 212 |
| Vitamin D3 indicator | 0.93 (0.49-1.77) | 0.827 | 0.91 (0.48-1.75) | 0.787 | 4,001 | 212 |
| Zoledronic acid indicator | 0.45 (0.20-1.00) | 0.049 | 0.50 (0.21-1.19) | 0.118 | 4,001 | 212 |
| Calcitonin indicator | 0.92 (0.63-1.34) | 0.656 | 0.90 (0.62-1.32) | 0.599 | 4,001 | 212 |
| Alendronate indicator | 1.82 (0.97-3.40) | 0.060 | 1.90 (1.04-3.48) | 0.036 | 4,001 | 212 |
| CCI 1 vs 0 | 1.08 (0.64-1.83) | 0.777 | 1.07 (0.62-1.82) | 0.816 | 4,001 | 212 |
| CCI >=2 vs 0 | 2.91 (1.54-5.50) | 0.001 | 2.58 (1.36-4.92) | 0.004 | 4,001 | 212 |
| Proximal humerus vs wrist | 1.18 (0.54-2.54) | 0.678 | 1.20 (0.55-2.58) | 0.649 | 4,001 | 212 |
| Thoracic vertebra vs wrist | 2.04 (1.16-3.60) | 0.014 | 2.06 (1.17-3.62) | 0.012 | 4,001 | 212 |
| Lumbar vertebra vs wrist | 1.83 (1.07-3.14) | 0.028 | 1.81 (1.07-3.08) | 0.027 | 4,001 | 212 |
| Hip fracture vs wrist | 1.30 (0.74-2.29) | 0.362 | 1.27 (0.71-2.26) | 0.415 | 4,001 | 212 |

Note. Both models used the identical complete-case cohort, covariates, target-event definition, and 212 first recorded hospitalized refracture events. HR, hazard ratio; sHR, subdistribution hazard ratio; CI, confidence interval; BMI, body mass index; FLS, Fracture Liaison Service; CCI, Charlson Comorbidity Index.

**Supplementary Table S4. Multivariable Cox model for all-cause mortality**

| **Variable** | **HR** | **95% CI** | **P value** | **Model N** | **Deaths, n** |
| --- | --- | --- | --- | --- | --- |
| Age per 10 years | 3.08 | 2.72-3.49 | <0.001 | 4,001 | 373 |
| Male sex | 1.55 | 1.25-1.92 | <0.001 | 4,001 | 373 |
| BMI, kg/m2 | 0.96 | 0.93-0.99 | 0.015 | 4,001 | 373 |
| Recorded FLS use | 1.22 | 0.72-2.08 | 0.457 | 4,001 | 373 |
| Recorded hypertension history | 1.21 | 0.94-1.55 | 0.139 | 4,001 | 373 |
| Calcium indicator | 1.07 | 0.85-1.34 | 0.573 | 4,001 | 373 |
| Alfacalcidol indicator | 1.05 | 0.83-1.32 | 0.688 | 4,001 | 373 |
| Vitamin D2 indicator | 1.00 | 0.71-1.40 | 0.983 | 4,001 | 373 |
| Vitamin D3 indicator | 1.05 | 0.69-1.60 | 0.830 | 4,001 | 373 |
| Zoledronic acid indicator | 0.96 | 0.56-1.63 | 0.875 | 4,001 | 373 |
| Calcitonin indicator | 1.17 | 0.89-1.53 | 0.258 | 4,001 | 373 |
| Alendronate indicator | 0.81 | 0.47-1.38 | 0.428 | 4,001 | 373 |
| CCI 1 vs 0 | 1.50 | 1.10-2.03 | 0.010 | 4,001 | 373 |
| CCI >=2 vs 0 | 2.08 | 1.30-3.33 | 0.002 | 4,001 | 373 |
| Proximal humerus vs wrist | 0.98 | 0.39-2.45 | 0.965 | 4,001 | 373 |
| Thoracic vertebra vs wrist | 1.32 | 0.66-2.67 | 0.432 | 4,001 | 373 |
| Lumbar vertebra vs wrist | 1.61 | 0.84-3.11 | 0.154 | 4,001 | 373 |
| Hip fracture vs wrist | 2.07 | 1.09-3.93 | 0.025 | 4,001 | 373 |

Note. The complete-case model included 4,001 patients and 373 deaths. The global Schoenfeld-residual test was not significant (p=0.216). Estimates are observational associations and are not interpreted as causal treatment effects. HR, hazard ratio; CI, confidence interval; BMI, body mass index; FLS, Fracture Liaison Service; CCI, Charlson Comorbidity Index.

**Supplementary Table S5. Analysis cohort, first-event counts, and observed death-after-refracture transitions**

| **Measure** | **Value** | **Definition or interpretation** |
| --- | --- | --- |
| Source cohort | 4,782 | All unique patients in the Clinical Variables Table. |
| Deaths <30 days after discharge | 31 | Excluded from the primary long-term cohort; all occurred within 0-26 days. |
| Death exactly on day 30 | 1 | Retained because the primary rule was Survivaltime >=30 days. |
| Primary long-term cohort | 4,751 | Patients with Survivaltime >=30 days from discharge. |
| First recorded hospitalized refracture as first event | 258 | Defined using Refracture >0 and the locked corrected first-event rule. |
| Death before recorded refracture | 487 | Observed competing deaths within the common refracture window. |
| Censored before either first event | 4,006 | No observed target or competing event before the common horizon. |
| Primary-cohort time-zero refracture | 1 | Retained and assigned 0.5 day for continuous-time models. |
| Primary-cohort same-day refracture and death | 1 | Refracture first; death placed 0.5 day later in multistate data. |
| Observed death on the refracture administrative boundary | 1 | Classified as competing death in the corrected rule. |
| No-refracture deaths after the refracture horizon | 6 | Censored at the refracture horizon in the primary competing-risk analysis. |
| Complete-case regression cohort | 4,001 | Identical cohort used for cause-specific Cox and Fine-Gray models. |
| Complete-case first-refracture events | 212 | Includes the source-recorded time-zero event after assignment to 0.5 day. |
| Refracture followed by death during full available follow-up | 53 | Observed patient sequences, not a state probability. |
| Refracture followed by death within 5 years | 47 | Observed patient sequences within 5 years. |
| Refracture-to-death transitions in the common-horizon model | 52 | Observed transitions contributing to the primary Aalen-Johansen analysis. |
| Estimated probability of death after refracture at 5 years | 1.88% (1.30-2.45) | Aalen-Johansen state-occupation probability, not 47/4,751. |
